# The appropriateness of initial intravenous empirical antibiotic consumption guided by digital antimicrobial stewardship tool: a correlation with the usage of antibiotics from the access and watch groups in ten Indonesian hospitals

**DOI:** 10.3389/fdgth.2026.1862829

**Published:** 2026-07-29

**Authors:** Ronald Irwanto Natadidjaja, Widyawati Lekok, Triyoko Septio Marja, Hadianti Adlani, Aziza Ariyani, Sri Mulyani, Siti Rahmah, Raymond Adianto, Yenny Yenny, Meutia Atika Faradilla, Jihan Samira

**Affiliations:** 1 Faculty of Medicine, Universitas Trisakti, Jakarta, Indonesia; 2 Trisakti – RASPRO Indonesia Antimicrobial Stewardship (TRIASE) Learning Center, Jakarta, Indonesia; 3 RASPRO Indonesia Study Group for Antimicrobial Stewardship and Resistance, Jakarta, Indonesia; 4 Trisakti School of Management, Jakarta, Indonesia; 5 Hermina Hospital Group, Jakarta, Indonesia; 6 Faculty of Medicine, Universitas Islam Negeri Syarif Hidayatullah, Banten, Indonesia

**Keywords:** antibiotic, appropriateness, defined daily dose, digital, empirical, intravenous, stewardship

## Abstract

**Background:**

RASPRO Indonesia Study Group for Antimicrobial Stewardship & Resistance has developed a digital antimicrobial stewardship (ASP) tool known as the e-RASPRO, which may provide a guideline for clinical decision on antibiotic prescribing for bacterial infection cases by integrating the WHO AWaRe (Access, Watch, Reserve) classification.

**Methods:**

A multicenter retrospective correlational study was conducted using aggregated monthly hospital antibiotic prescribing data. A correlation test was conducted between the mean percentage on the appropriateness of initial intravenous empirical antibiotic consumption and the mean Defined Daily Dose (DDD) of antibiotics within a year (January to December 2024) in 10 Indonesian hospitals after the socialization of e-RASPRO tool between 2022 and 2024 in four major infections.

**Results:**

There was a significant positive correlation between the mean of percentages on appropriateness of initial intravenous empirical antibiotic consumption in type I risk stratification guided by e-RASPRO tool and the mean DDD/100 patient-days of intravenous Penicillin as Access group antibiotic (*r* = 0.598, 95% CI 0.021–0.873; *p* = 0.041) and a significant negative correlation with the mean DDD/100 patient-days of intravenous third-generation Cephalosporins as Watch group antibiotic (*r* = −0.612, 95% CI −0.878 to −0.044; *p* = 0.035) after removed linear temporal trends.

**Conclusion:**

Appropriateness of initial intravenous empirical antibiotic consumption in type I and II risk stratification guided by e-RASPRO tool was correlated with increased consumption of Penicillin as an Access group antibiotic and decreased consumption of third-generation Cephalosporins as a Watch group antibiotic. This study did not describe a causal relationship between the use of the e-RASPRO tool and antibiotic consumption in the hospital.

## 1 Introduction

World Health Organization (WHO) has classified antibiotics into Access, Watch, and Reserve categories. It is expected that the target of antibiotic prescribing from the Access group in various countries may reach at least 60% (1, 2). Supports from hospital management, human resources and other supporting tools are absolutely necessary in order to achieve the WHO target. The implementation of the digital Antimicrobial Stewardship (ASP) may provide good results on the prescribing of antimicrobial agents and it may reduce Defined Daily Dose (DDD) of antibiotics; however, the appropriate digital model has yet to be determined (3–5). The WHO Access, Watch, Reserve (AWaRe) classification can be used as a guideline in consuming antimicrobial agents (6). Based on the current regulation in Indonesia, antibiotics from the Access category can be prescribed without any pre-authorization although appropriate indication would be necessary; while those from the Watch and Reserve groups must be given based on pre-authorization and supervision by the Antimicrobial Stewardship team, infection control consultants and the hospital's Antimicrobial Resistance Control Committee (ARCC) (7). On the other hand, the management and technology of antimicrobial prescribing, as well as the implementation and operational monitoring on the consumption of antimicrobial agents, are often overlooked a part of disparities in the development of National Action Plans (NAPs) (8).

A small-scale survey conducted in 2019, which involved 156 hospital respondents in Indonesia, found that 114 respondents reported that they had not implemented antimicrobial resistance control measures in their hospitals, largely due to the lack of a clear management framework for implementing such measures (9). Another larger survey conducted in Iran from 2022 to 2023 reported that 63% of antibiotics prescribed to outpatients belonged to the WHO Watch category (10). The RASPRO Indonesia Study Group for Antimicrobial Stewardship & Resistance views this as a gap in management, antimicrobial prescribing technology, as well as implementation and operational monitoring that could lead to the failure to implement the ASP in a hospital in Indonesia.

In order to narrow the gap, the RASPRO Indonesia Study Group for Antimicrobial Stewardship and Resistance has developed a Digital ASP tool known as the e-RASPRO, which features digital antibiotic guidelines that classify patients into Types I, II, and III risk stratification based on severity, immune status and medical history. The tool has also integrated the WHO AWaRe antibiotic classification by considering local microorganism pattern (if it is available at the hospital) as well as various considerations that have been evaluated through peer expert review at the hospital. By implementing the tool, it is expected that it can serve as one of modalities to provide a guideline for clinicians as well as monitoring, evaluating and reporting on the administration of prophylactic, empirical, and definitive antibiotics. The Digital ASP e-RASPRO tool can also facilitate on-site consultations between doctors, pharmacists and the hospital's ASP team to coordinate the conduct of prospective pre-authorization audits regarding antibiotic use in the hospital.

This study is expected to serve as a preliminary study involving multiple hospitals with a substantial volume of prescriptions. It aims to examine the correlation between the mean percentage of appropriate initial intravenous empirical antibiotic use, based on the Antibiotic Guidelines of the Digital Antimicrobial Stewardship Program (ASP) e-RASPRO Tool for four major infections (intra-abdominal biliary infection, pneumonia, urinary tract infection, and skin and soft tissue infection), and the mean Defined Daily Dose (DDD) of intravenous antibiotics from the WHO Access and Watch categories in 2024 across 10 hospitals. We hypothesize that a higher rate of concordance with empirical prescribing guidelines for initial intravenous antibiotics in these four major infections will be associated with increased consumption of Access antibiotics and decreased consumption of Watch antibiotics. Nevertheless, we recognize that this study has not clearly explained the cause-and-effect relationship and remains limited to the use of initial intravenous empirical antibiotics, which is associated with antibiotic DDD within a year period of time.

## 2 Methods

### 2.1 Early socialization on implementing digital antimicrobial stewardship e-RASPRO tool

Socialization and training program on the Digital ASP e-RASPRO tool was conducted gradually by implementing the digital guidelines for empirical antibiotic therapy at 10 hospitals in Indonesia from 2022 to 2024. The Digital ASP e-RASPRO tool would guide clinicians in deciding on the administration of empirical antibiotics and help hospitals to monitor the appropriateness of the administered antibiotics whether it had been complied with current guidelines.

At these ten hospitals, the digital guidelines for empirical antibiotic therapy outlined in the Digital ASP e-RASPRO tool had been implemented, as agreed upon through peer expert reviews at each hospital. In this setting, patients with bacterial infections who would receive empirical antibiotics and would be admitted to an inpatient ward were divided into three risk stratification groups based on their disease severity, immune status, and medical history (5, 11):

- **Type I risk stratification group** is a group of patients with mild bacterial infections, whether immunocompetent or immunocompromised, whose infections do not meet the criteria for Type II and III risk stratification groups; in other words, they are at lower risk of infection with multidrug-resistant (MDR) bacteria.
  Based on the digital guidelines for empirical antibiotic therapy approved through peer expert review by each hospital, in the e-RASPRO tool, this group may receive empirical antibiotics from the Access category, such as Amoxicillin Clavulanate, Ampicillin Sulbactam (Penicillin class - Access) with or without a combination of Amikacin/Gentamicin (Aminoglycoside class - Access). In this risk stratification group, the Quinolone group, which falls under the Watch category, is used in cases of beta-lactam allergy or based on specific clinical considerations.
- **Type II risk stratification group** is a group of patients with mild bacterial infection with the following characteristics: ((immunocompromised AND/OR uncontrolled diabetes mellitus) + (history of antibiotic use within ≤ 90 days AND/OR history of hospitalization at a healthcare facility for 48 h or more (≥ 48 h) but less than 90 days ago (≤ 90 days) AND/OR history of medical device use within ≤ 90 days ago). The group of patients with such characteristics are at risk for infection by MDR bacteria—Extended-Spectrum Beta-Lactamases (ESBLs) (11–17). This group of patients may be prescribed anti-ESBL antibiotics from the Access – Watch category. Based on the digital guidelines for empirical antibiotic therapy approved through peer expert review by each hospital, in the e-RASPRO tool, this group may receive empiric antibiotic treatment of Carbapenem Sparing Agent in order to eradicate ESBLs involving the Access antibiotics, i.e., the combination of Ampicillin Sulbactam/Amoxicillin Clavulanate (Penicillin class - Access) must be combined with Amikacin/Gentamicin (Aminoglycosides class - Access). In this risk stratification group, the Quinolone group, which falls under the Watch category, is used in cases of beta-lactam allergy or based on specific clinical considerations.
- **Type III risk stratification group** is a group of patients with severe/threatening bacterial infection AND/OR patients with Healthcare Associated Infections (HAIs) AND/OR ((Immunocompromised AND/OR uncontrolled diabetes mellitus) + (history of antibiotic use of ≤ 30 days ago AND/OR history of hospitalization at a healthcare facility for 48 h or more (≥ 48 h) but less than 30 days ago (≤ 30 days) AND/OR history of medical device use within ≤ 30 days ago). This group of patients with these characteristics is at high risk of mortality and is also at risk of infection by ESBL bacteria and other multidrug-resistant pathogens, such as methicillin-resistant *Staphylococcus aureus* (MRSA), multidrug-resistant *Pseudomonas spp.,* and others (11, 18–28). Based on the digital guidelines for empirical antibiotic therapy approved by peer experts at each hospital using the e-RASPRO tool, this group may receive antibiotics in the ‘Watch’ category, with or without combination therapy using antibiotics in the ‘Access’ category: Meropenem/Imipenem/Doripenem (Carbapenem class -Watch) with or without a combination of Amikacin/Gentamicin (Aminoglycoside class -Access). In cases of suspected MRSA infection, anti-MRSA antibiotics may be administered. The use of these antibiotics must undergo a pre-authorization process and prospective audit by the hospital's ASP Team. In this risk stratification group, Quinolone group, which falls under the ‘Watch’ category, are used in cases of carbapenem allergy or based on specific clinical considerations.

The Digital ASP e-RASPRO tool would notify doctors, pharmacists, and nurses regarding the category of antibiotics to be administered (Access, Watch, or Reserve). In accordance with national regulations in Indonesia, if the antibiotic to be used is categorized as Watch or Reserve, the pharmacy is required to consult with the hospital's ASP team, infection control consultant, and hospital ARCC for pre-authorization and prospective audit.

The Digital ASP e-RASPRO tool could be used to guide the selection of initial empirical antibiotics, guide subsequent changes or additions to empirical antibiotic therapy (if necessary), record definitive antibiotic therapy, guide the use of prophylactic antibiotics, issue notifications regarding prolonged antibiotic use, and provide physician notes for difficult-to-treat bacterial infection cases requiring comprehensive discussion. However, in the flowchart below, we have limited the scope to the system that guides physicians in determining the use of initial empirical antibiotics.

The following is a screenshot from the Digital ASP e-RASPRO tool showing the workflow for determining the use of initial empirical antibiotics (the first empirical antibiotics prescribed by a doctor when a patient is admitted to the hospital), using the digital empirical antibiotic guidelines on this device (5).

**Figure 1:**
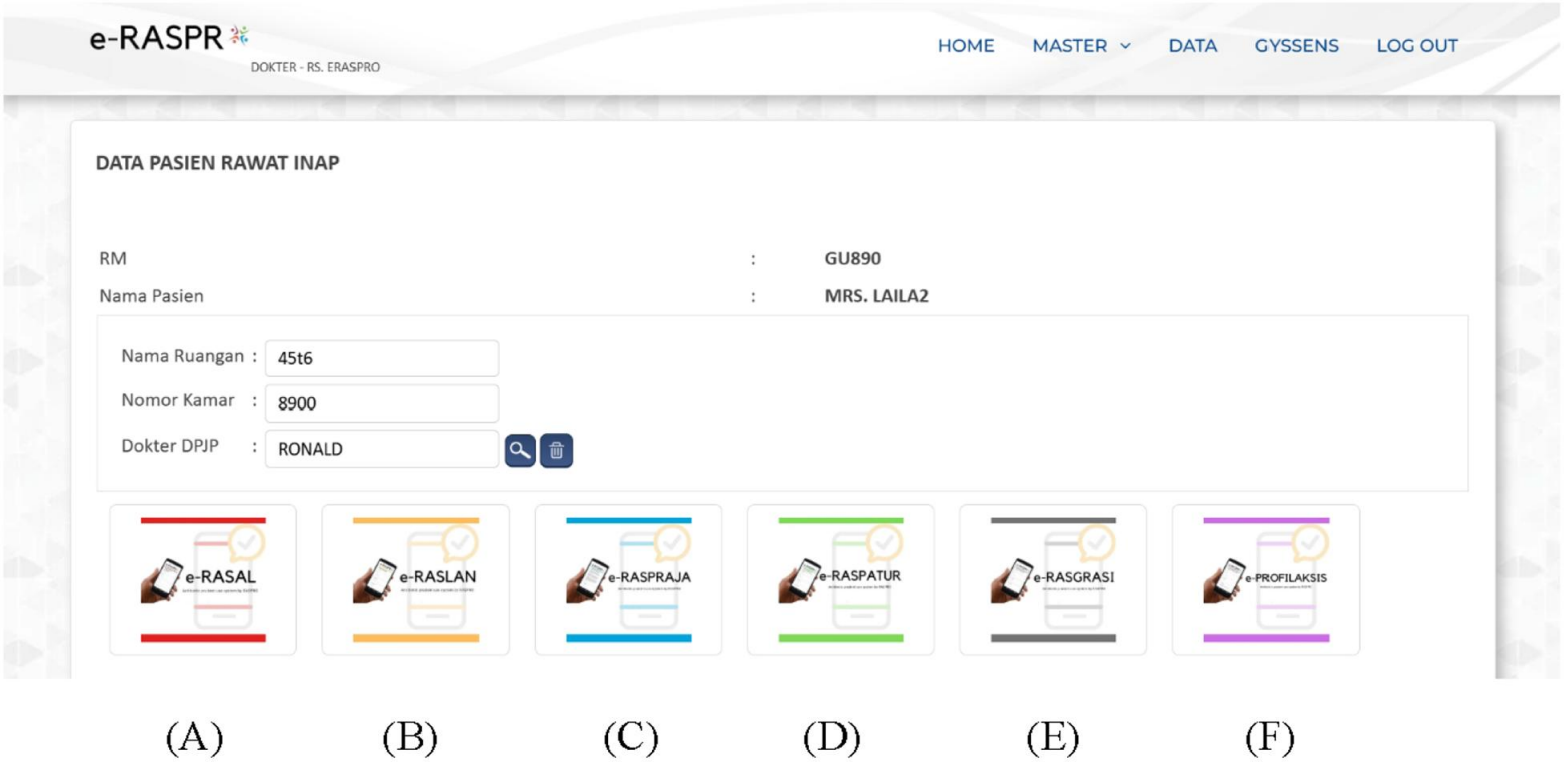
The doctor's screen serves as a guideline on prescribing antibiotic, reporting cases of prolonged antibiotic use and reporting cases of difficult-to-treat bacterial infections. (A) RASAL Box. (B) RASLAN Box. (C) RASPRAJA Box. (D) RASPATUR Box. (E) RASGRASI Box. (F) e-Prophylaxis Box.

The Digital ASP e-RASPRO tool is designed to guide and monitor physicians in prescribing antibiotics. Figure 1 shows boxes that can be selected by physicians for antibiotic use in daily practice for hospitalized patients. Box (A) was referred to as the RASAL Box. This box must be checked when the doctor decided to administer initial empirical antibiotics (intravenous or oral). The initial empirical antibiotic was the first antibiotic decided by the doctor to administer for a patient upon admission to the inpatient ward. Box (B) was referred to as the RASLAN Box. This box must be checked when the doctor decided to change the empirical antibiotic regimen during treatment, if necessary. The RASLAN box could be used as a guide for escalating (step-up) or de-escalating (step down) antibiotic treatment based on digital empirical guidelines and the doctor's clinical observations.

Box (C) was referred to as the RASPRAJA Box. This box must be checked and filled out when a doctor decided that there were specific indications requiring prolonged antibiotic administration (whether empirical or definitive). If antibiotic use exceeds a certain duration and the doctor failed to check the RASPRAJA Box, the hospital may issue an Automatic Stop Order (ASO).

Box (D) was called the RASPATUR Box. This box must be checked when a doctor prescribed a definitive antibiotic. Box (E) was called the RASGRASI Box. This box can be checked when a doctor would report difficult-to-treat bacterial infection cases, in which the patient had already received multiple changes of antibiotic treatment. When the RASGRASI Box was clicked, a notification would be sent to the pharmacist, and the difficult case could be submitted for a collaborative case review involving the Hospital's ARCC. Box (F) was called the e-Prophylaxis Box, which could be used to guide doctors in the administering prophylactic antibiotics.

**Figure 2:**
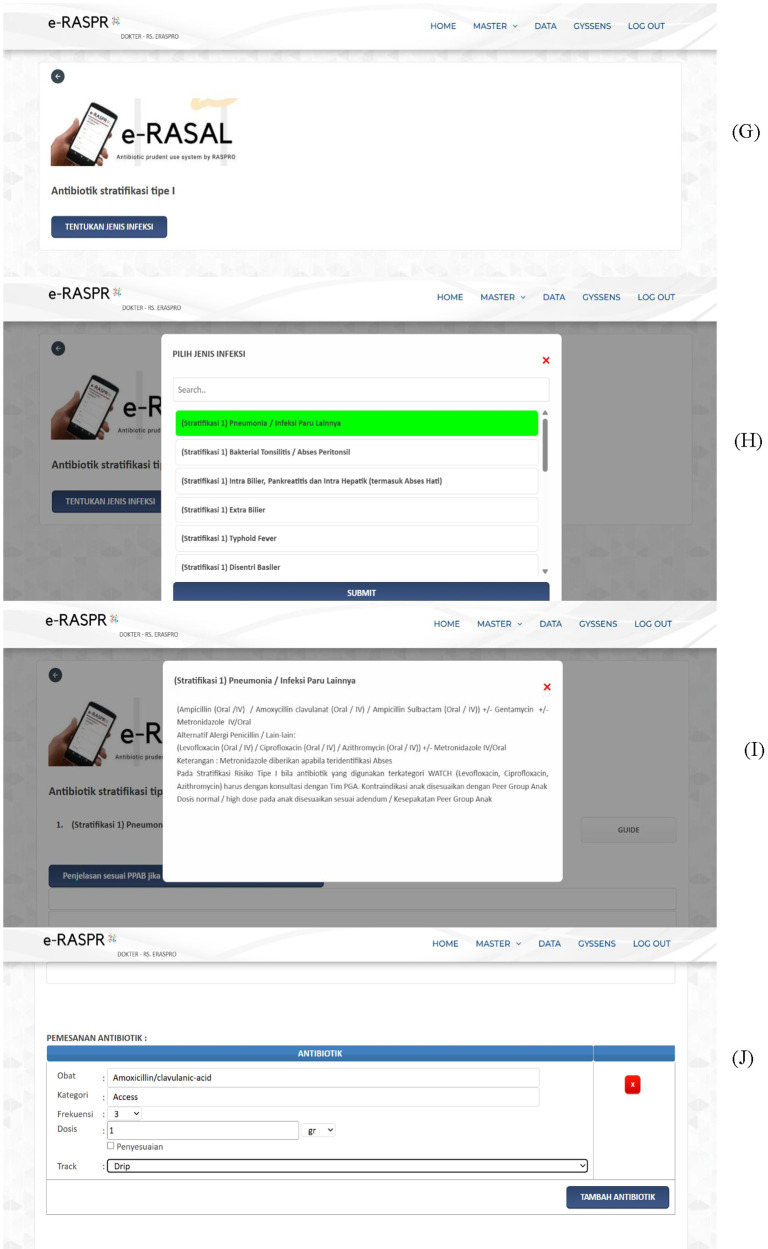
The doctor's screen showing workflow and guideline to determine initial empirical antibiotics. (G) Patient Risk Stratification Guided by e-RASPRO tool. (H) Determination of the site of bacterial infection by the physician in e-RASPRO tool. (I) Empirical antibiotic guidelines in e-RASPRO. (J) WHO AWaRe antibiotic category notifications in e-RASPRO.

After the doctor clicked the RASAL box (A) in Figure 1, the screen advanced to the next phase, as shown in Figure 2. Figure 2 illustrates how the Digital ASP e-RASPRO tool guided the physician in administering initial empirical antibiotics (intravenous or oral) when a patient was admitted to the hospital.

The doctor could input data about the patient's condition based on current clinical observations, including: (1) Severity of illness of the patient's infectious disease (Sepsis/Non-Sepsis, Febrile Neutropenia/Healthcare-Associated Infections/Life-Threatening Organ Perforation/Encephalopathy associated with severe bacterial infection), (2) Immune status (immunocompetent/immunocompromised), (3) The patient's medical history over the past 30 or 90 days (history of antibiotic consumption, history of hospitalization, and history of medical device use). the Digital ASP e-RASPRO tool would provide doctors with information regarding patient risk stratification in accordance with the various academic citations previously cited (G).

After that, the doctor could continue this workflow by clicking on the patient's site of bacterial infection (H). The digital empirical antibiotic guide would provide initial empirical antibiotic options that could be administered to the patient based on the patient's risk stratification. The empirical antibiotic guide was customized according to the guideline that had been agreed upon through a peer expert review at each hospital (I). The doctor could then select the initial empirical antibiotic, along with the dosage, frequency of administration, and route of administration. Through this screen, the doctor would also immediately receive information on whether the prescribed antibiotic fell under the Access, Watch, or Reserve category (J).

After the doctor submitted the initial empirical antibiotic prescription as shown in Figure 2, the workflow in this system would forward it to the pharmacist for review.

**Figure 3:**
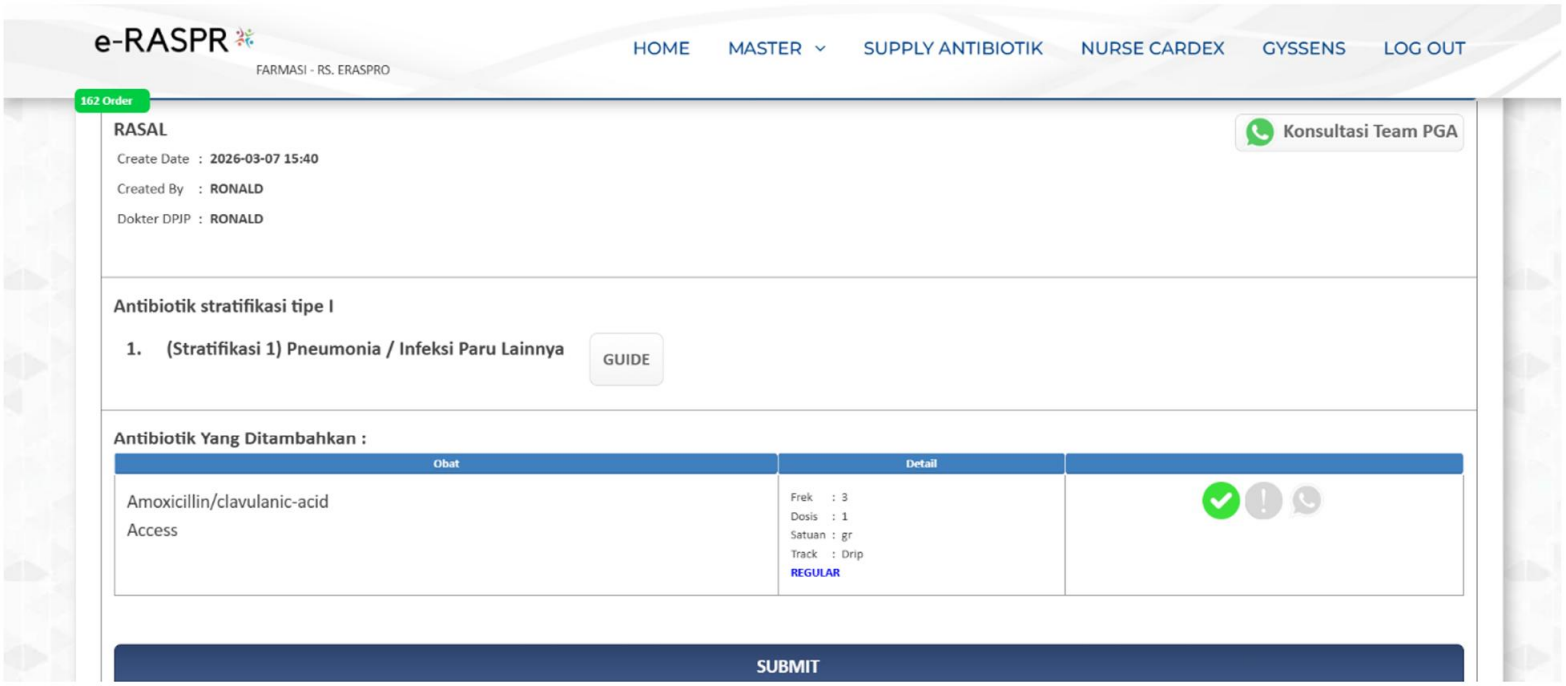
The pharmacist's screen to evaluate antibiotics that had been administered.

Figure 3 shows the pharmacist's screen, which was used to review the prescribed antibiotics. Through this screen, the pharmacist would be informed of various details, including: (1) Site of infection, (2) Patient risk stratification, (3) Appropriateness of antibiotic prescription based on the digital empirical guidelines (by clicking the ‘Guide’ button), (4) Type of antibiotic that had been administered, antibiotic category (Access/Watch/Reserve), (5) Dose, frequency and route of administration.

Pharmacists could click the phone icon in the upper-right corner to consult with the hospital's ASP Team on-site if the prescribed antibiotics did not comply with the guidelines AND/OR if the prescribed antibiotics fell under the Watch or Reserve categories. If no agreement had been reached regarding the use of antibiotics among the pharmacist, the hospital's ASP team, and the physician, but the antibiotics must be dispensed immediately for patient safety reasons, the pharmacist may issue a special notification through this system. These notifications could then be submitted to hospital management every three to six months (semester) to serve as a basis for evaluation and to determine future policies regarding the prudent use of antibiotics.

Once the pharmacist had completed the assessment of the initial empirical antibiotic prescription as shown in Figure 3, the pharmacist could submit the assessment and the workflow on the system would forward it to the nurse so that they could also monitor antibiotic use.

**Figure 4:**
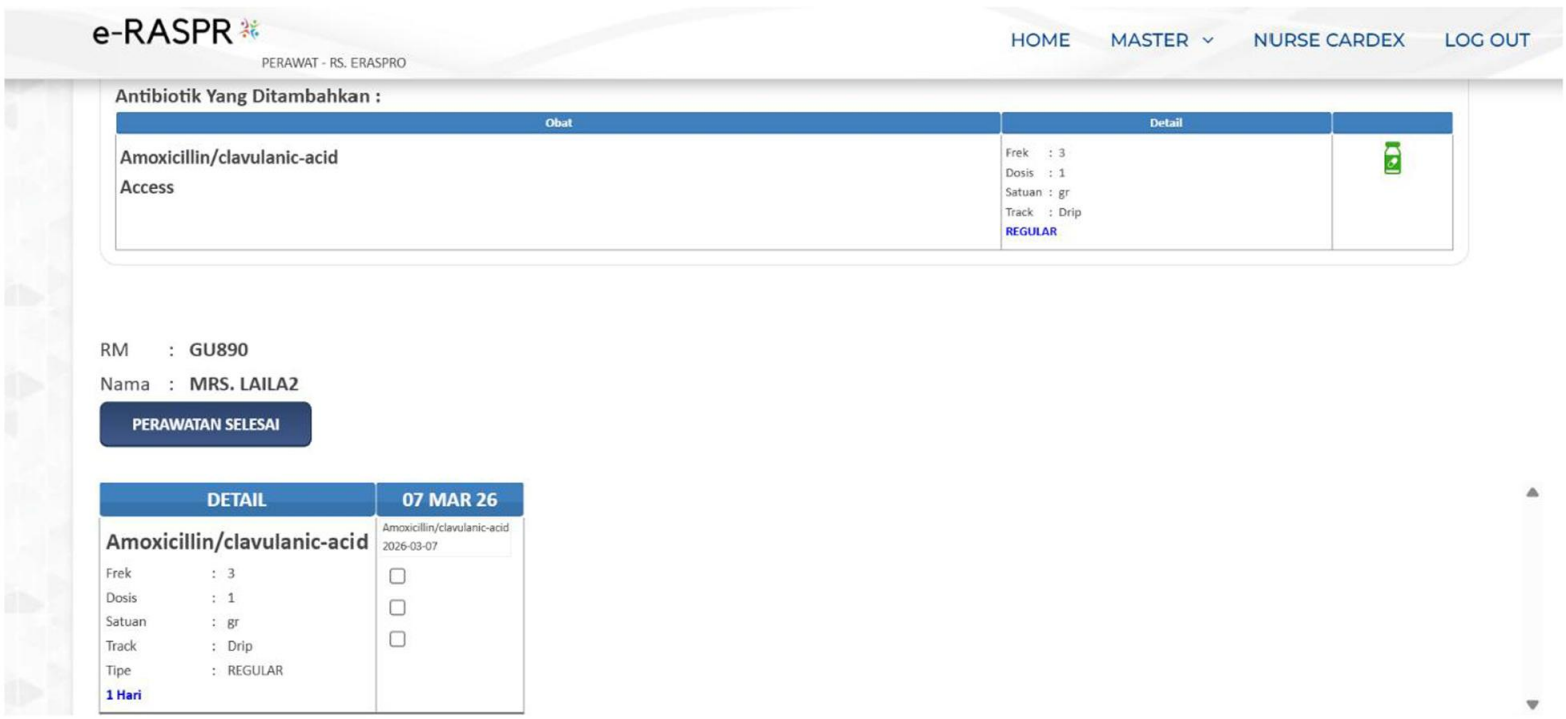
The nurse's screen to monitor antibiotic use.

Figure 4 shows the nurse's screen, in which the nurse could access various pieces of information, namely: (1) the site of the patient's infection, (2) the patient's risk stratification, (3) the appropriateness of antibiotic prescription according to the digital empirical guidelines (by clicking the ‘Guide’ button), (4) the type of antibiotic that had been administered and the antibiotic category (Access/Watch/Reserve), (5) The dose, frequency and route of administration of antibiotics. Nurses and pharmacists could also monitor how many days the antibiotics were administered. If a doctor prescribed long-term antibiotics, the nurse and/or pharmacist could remind the doctor to document the indication for long-term antibiotic use in the RASPRAJA Box (Figure 1F), because if the indication had not been clearly documented through that box, the hospital might enforce Automatic Stop Order (ASO).

### 2.2 Obtaining and analyzing data

This was a multicenter retrospective correlational study using aggregated monthly hospital antibiotic prescribing data. The data were retrospectively collected from inpatient units at 10 hospitals between January and December 2024 using the Digital Antimicrobial Stewardship Program (ASP) e-RASPRO tool. The inclusion criteria comprised initial intravenous empirical antibiotics prescribed for all inpatients, both adult and pediatric, including ICU patients, for four major infections. The use of empirical combination therapy initiation was included in the calculation and was declared appropriate if the combination therapy was in accordance with the antibiotic guidelines in e-RASPRO. In accordance with the study title and primary objective, change of antibiotic from the initial antibiotic or a change of antibiotic to the definitive antibiotic that occurred during hospitalization were excluded. The same empirical antibiotic guideline was in effect at all 10 hospitals, as agreed upon by each hospital's peer expert review committee. Data were retrieved in early 2025, which included the following:

1. Percentage of Appropriate Use of Initial Empirical Intravenous Antibiotics (the first empirical intravenous antibiotic administered upon a patient's admission to the inpatient ward) for the four most common infections (the ‘4 Major’ infections), i.e., Intrabiliary Infections, Pneumonia, Urinary Tract Infection and Pyelonephritis, and Soft Tissue Infection at 10 hospitals between January and December 2024.

The four major infections selected were those that deemed to be highly prevalent in the 10 hospitals, which had been studied during 2024. The consumption of a single initial intravenous empirical antibiotic was categorized as appropriate if it complied with the guidelines in the Digital ASP e-RASPRO tool. The use of a combination of initial intravenous empirical antibiotics was categorized as appropriate if both complied with the guideline in the Digital ASP e-RASPRO tool.

(1) Mean DDD of Intravenous Antibiotics. In accordance with Indonesian national regulations, DDD refers to the Defined Daily Dose/100 patient-days, hereafter termed DDD.DDD/100patientdays= $\frac{\mathrm{Total}\;\mathrm{Antibiotic}\;\mathrm{Used}\;(\mathrm{gram})\;/\;\mathrm{WHO}\;\mathrm{DDD}}{\mathrm{Total}\;\mathrm{Length}\;\mathrm{of}\;\mathrm{Stay}\;(\mathrm{LOS})}\times 100$.

Data about mean DDD for the five largest classes of intravenous antibiotics were collected and calculated from 10 hospitals, specifically for the Access Group (Penicillins and Aminoglycosides) and the Watch Group (third-generation Cephalosporins, Quinolone, and Carbapenem group) between January and December 2024 to observe the trends of antibiotic consumption during that year.

Afterwards, the percentage on the appropriateness of initial intravenous empirical antibiotic consumption based on the digital antibiotic guidelines was described according to patient risk stratification groups for the 4 major infections, as well as the mean DDD of the five classes intravenous antibiotics between the first semester (January to June 2024) and 2nd semester (July to December 2024) during the implementation of the Digital ASP e-RASPRO tool. A correlation analysis was conducted between the mean percentage on the appropriateness of initial empirical intravenous antibiotic consumption based on the digital antibiotic guidelines for the 4 major infections and the mean DDD of intravenous antibiotics in 2024.

All patient-level identifiers were removed and anonymized before aggregation and analysis were performed. Data processing, discussions, and various verifications were conducted from mid- to late 2025. The appropriateness of initial intravenous empirical antibiotics consumption and the DDD of intravenous antibiotics were verified by pharmacists from each hospital using the Digital ASP e-RASPRO tool. All data extracted from the Digital ASP e-RASPRO tool was then re-verified by the ARCC and peer experts from the ten hospitals. Data analysis and manuscript writing began in early 2026 under the supervision of the various parties involved in this study.

## 3 Results

The results of this study are presented in the five tables provided by the researchers. All data were obtained in 2024 from 10 hospitals.

Table 1 shows the total number of initial empirical intravenous antibiotic prescriptions for the four major infections at 10 hospitals, which was recorded in the Digital ASP e-RASPRO tool, which reached 20,907 prescriptions during 2024. During the first semester, about 4,886 prescriptions were documented; while in the second semester, 16,021 prescriptions were recorded. There was an increase in the mean percentage on the appropriateness of initial intravenous empirical antibiotic consumption in the combined type I and II risk stratification group from 65.1% in the first semester to 90.6% in the second semester; while for those in the type III risk stratification group, there was an increase from 63.8% in the first semester to 86.2% in the second semester.

**Table 1:** Description on mean percentage of appropriateness of initial intravenous empirical antibiotic consumption in two groups (combined type I and II risk stratification group and type III group) based on the digital ASP e-RASPRO tool guidelines for the four major infections between the first and second semesters in 2024 at 10 hospitals.

Initial intravenous empirical antibiotics for the four major infection	The 1st semester January to June 2024		The 2nd semester July to December 2024		Total
	*n*	Mean Percentage of Appropriateness (x100%)	*n*	Mean Percentage of Appropriateness (x100%)	
Total Prescription	4,886		16,021		20,907
Type I + II Risk Stratification		0.651 (0.302)		0.906 (0.018)	–
Type III Risk Stratification		0.638 (0.297)		0.862 (0.045)	–

Values in parentheses are standard deviations.

Table 2 shows a sharp increase in the mean DDD for Penicillin-class antibiotics and a decrease for third-generation Cephalosporins. The DDD for Penicillin-class antibiotics increased from 30.648 (7.245) to 47.879 (7.374), while the DDD for intravenous third-generation Cephalosporins consumption decreased from 16.369 in the first semester to 13.071 in the second semester. There was an increase in DDD for aminoglycosides, Quinolone and Carbapenem, but it was not as significant as the increase in DDD for Penicillin.

**Table 2:** Description of the mean daily dose (DDD) of intravenous antibiotics in the first and second semesters following the implementation of the digital ASP e-RASPRO tool in 2024 at 10 hospitals.

Antibiotic class	The 1st semester January to June 2024	The 2nd semester July to December 2024
	Mean DDD	Mean DDD
Penicillin	30.648 (7.245)	47.879 (7.374)
Aminoglycosides	9.807 (0.585)	9.906 (1.325)
third-generation Cephalosporins	16.369 (1.967)	13.071 (1.391)
Quinolone	27.963 (2.941)	31.536 (1.485)
Carbapenem	6.456 (1.893)	8.619 (3.204)

Values in parentheses are standard deviations.

Table 3 shows a significant correlation between the appropriateness of initial intravenous empirical antibiotic consumption in type I and II risk stratification with the use of intravenous penicillin and third-generation Cephalosporins. After detrending via standardized residuals to remove linear temporal trends, significant positive and negative correlations persisted between the mean percentage on appropriate initial intravenous empirical antibiotics in the combined type I and II risk stratification group and the mean DDD of intravenous Penicillin (*r* = 0.598, 95% CI 0.021–0.873; *p* = 0.041) and intravenous third-generation Cephalosporins (*r* = −0.612, 95% CI −0.878 to −0.044; *p* = 0.035). For both antibiotics, the 95% confidence intervals for raw and detrended analyses excluded zero, indicating that month-to-month changes in appropriateness were associated with concurrent changes in antibiotic use independent of overall secular trends.

**Table 3:** Description and correlation of mean percentage on the appropriateness of initial intravenous empirical antibiotic consumption in combined types I and II risk stratification group based on antibiotic guidelines of the digital ASP e -RASPRO tool for the four major infections overall with the mean DDD of intravenous antibiotics from the access and watch categories in 2024 at 10 hospitals.

			Access				Watch					
Months	Appropriateness (x 100%)		Penicillin		Aminoglycoside		third-generation Cephalosporins		Quinolone		Carbapenem	
			Mean DDD		Mean DDD		Mean DDD		Mean DDD		Mean DDD	
		*r*		0.784		0.221		−0.743		−0.159		−0.077
		95% CI^a^		0.376 to 0.938		−0.402 to 0.704		−0.923 to −0.287		−0.668 to 0.451		−0.620 to 0.512
		*p*		0.003		0.490		0.006		0.622		0.813
		Detrended *r*^a^		0.598		0.184		−0.612		−0.203		−0.102
		95% CI Detrended *r*^a^		0.021 to 0.873		−0.439 to 0.689		−0.878 to −0.044		−0.701 to 0.416		−0.634 to 0.491
		Detrended *p*^a^		0.041		0.568		0.035		0.527		0.755
January	0.3465		22.98		8.89		19.86		32.52		7.83	
February	0.2727		23.34		9.81		16.65		30.17		9.49	
March	0.5455		27.42		9.92		16.42		27.75		4.87	
April	0.9286		35.26		10.38		15.37		25.83		5.85	
May	0.8947		33.84		9.43		15.97		27.06		6.21	
June	0.9184		41.06		10.42		13.94		24.47		4.49	
July	0.8887		40.74		10.29		13.89		31.03		5.11	
August	0.9152		43.99		11.03		14.43		34.24		10.70	
September	0.9203		45.54		10.18		14.58		29.91		10.85	
October	0.8862		46.50		10.97		12.09		31.64		10.83	
November	0.8946		48.55		7.45		12.07		31.75		10.30	
December	0.9304		61.95		9.53		11.36		30.65		3.93	

*r*, Pearson correlation coefficient; CI, confidence interval; *p*, two-tailed nominal *p*-value. 95% CI calculated via Fisher Z transformation: Z = 0.5 × ln[(1 + *r*)/(1−*r*)]; SE = 1/√(*n*−3) = 0.333; 95% CI for Z = Z ± 1.96 × SE; back-transformed to *r*. Computed using SPSS v29 CORRELATIONS/PRINT = TWOTAIL NOSIG CI (95). Detrended *r* = Pearson correlation of standardized residuals [ZRESID] from separate linear regressions of each variable on sequential month [1–12], SPSS v29 REGRESSION/SAVE ZRESID. Correlation analysis was based on monthly aggregate observations, not individual prescriptions. Number of monthly observations correlated with DDD = 12 months. No adjustment for multiple comparisons was applied. All analyses are exploratory.

a Assisted by artificial Intelligence with data input and analysis were verified by the authors supported by statistician.

No significant correlation was found between the mean percentage on appropriate initial intravenous empirical antibiotics in the combined type I and II risk stratification group and the mean DDD of intravenous Quinolone, even though the Quinolone group was used as an alternative antibiotic for this group.

Table 4 shows an insignificant correlation between the variables tested. After detrending via standardized residuals to remove linear temporal trends, correlations between the mean percentage on appropriate initial intravenous empirical antibiotics in the type III risk stratification group and the mean DDD of intravenous remained non-significant and close to zero for both intravenous Quinolone (*r* = 0.081, 95% CI −0.576–0.672; *p* = 0.825) and Carbapenem (*r* = −0.177, 95% CI −0.722–0.507; *p* = 0.625).

**Table 4:** Description and correlation of the mean percentage on the appropriateness of initial intravenous empirical antibiotics in the type III risk stratification group based on the antibiotic guidelines of the digital ASP e –RASPRO tool for the four major infections overall with the mean DDD of intravenous quinolone and carbapenem in 2024 at 10 hospitals.

				Watch		
Months	Appropriateness (x 100%)		Quinolone		Carbapenem	
			Mean DDD		Mean DDD	
		*r*		0.128		−0.212
		95% CI^a^		−0.544 to 0.698		−0.740 to 0.480
		*p*		0.724		0.557
		Detrended *r*^a^		0.081		−0.177
		95% CI Detrended *r*^a^		−0.576 to 0.672		−0.722 to 0.507
		Detrended *p*^a^		0.825		0.625
January	–		32.52		7.83	
February	0.2500		30.17		9.49	
March	–		27.75		4.87	
April	0.5625		25.83		5.85	
May	0.8580		27.06		6.21	
June	0.8831		24.47		4.49	
July	0.8826		31.03		5.11	
August	0.8628		34.24		10.70	
September	0.8284		29.91		10.85	
October	0.8079		31.64		10.83	
November	0.8554		31.75		10.30	
December	0.9360		30.65		3.93	

*r*, Pearson correlation coefficient; CI, confidence interval; p, two-tailed nominal *p*-value. 95% CI calculated via Fisher Z transformation: Z = 0.5 × ln[(1 + *r*)/(1−*r*)]; SE = 1/√(*n*−3) = 0.333; 95% CI for Z = Z ± 1.96 × SE; back-transformed to r. Computed using SPSS v29 CORRELATIONS/PRINT = TWOTAIL NOSIG CI (95). Detrended *r* = Pearson correlation of standardized residuals [ZRESID] from separate linear regressions of each variable on sequential month [1–12], SPSS v29 REGRESSION/SAVE ZRESID. Correlation analysis was based on monthly aggregate observations, not individual prescriptions. Missing data for January and March was due to administrative issues during data sorting, which suggested a lack of data purity. Therefore, it was decided to exclude data for those months. Number of monthly observations correlated with DDD = 10 months. No adjustment for multiple comparisons was applied. All analyses are exploratory.

a Assisted by artificial Intelligence with data input and analysis were verified by the authors supported by statistician.

Table 5 shows various correlations between appropriateness on initial empirical intravenous antibiotic consumption and DDD of antibiotics in four major infections grouped by risk stratification. In type I + II risk stratification patients, prescribing appropriateness was significantly positively correlated with intravenous Penicillin consumption for pneumonia (*n* = 12; *r* = 0.785, 95% CI 0.377–0.938; *p* = 0.002), urinary tract infection and pyelonephritis (*n* = 10; *r* = 0.654, 95% CI 0.010–0.913; *p* = 0.040), and soft tissue infection (*n* = 11; *r* = 0.623, 95% CI 0.052–0.888; *p* = 0.041). Significant inverse correlations with Watch group antibiotics were observed for intravenous third-generation Cephalosporins in pneumonia (*n* = 12; *r* = −0.719, 95% CI −0.915 to −0.236; *p* = 0.008), urinary tract infection and pyelonephritis (*n* = 10; *r* = −0.783, 95% CI −0.949 to −0.316; *p* = 0.007), and soft tissue infection (*n* = 11; *r* = −0.760, 95% CI −0.931 to −0.309; *p* = 0.007), and for Carbapenem in intrabiliary infection (*n* = 9; *r* = −0.699, 95% CI −0.932 to −0.071; *p* = 0.036).

**Table 5:** The correlation between the mean percentage of the appropriateness on initial empirical intravenous antibiotic consumption for each of the four major infections based on the digital antibiotic guidelines of ASP e-RASPRO system and the mean DDD of various classes of intravenous antibiotics in 2024 at 10 hospitals.

Types of Infection	Appropriateness			Penicillin			Aminoglycoside			third-generation Cephalosporins			Quinolone			Carbapenem	
		*n*	*r*	95% CI^a^	*p*	*r*	95% CI^a^	*p*	*r*	95% CI^a^	*p*	*r*	95% CI^a^	*p*	*r*	95% CI^a^	*p*
Intrabiliary Infection	Type I + II Risk Stratification	9	0.034	−0.645 to 0.682	0.930	−0.256	−0.784 to 0.483	0.507	0.051	−0.634 to 0.691	0.897	−0.650	−0.920 to 0.021	0.058	−0.699	−0.932 to −0.071	0.036
	Type III Risk Stratification	8										0.608	−0.166 to 0.921	0.110	0.075	−0.660 to 0.733	0.860
Pneumonia	Type I + II Risk Stratification	12	0.785	0.377 to 0.938	0.002	0.191	−0.428 to 0.689	0.553	−0.719	−0.915 to −0.236	0.008	−0.108	−0.637 to 0.487	0.738	−0.026	−0.589 to 0.555	0.936
	Type III Risk Stratification	10										0.166	−0.517 to 0.716	0.647	−0.185	−0.726 to 0.502	0.608
Urinary Tract Infection and Pyelonephritis	Type I + II Risk Stratification	10	0.654	0.010 to 0.913	0.040	0.369	−0.333 to 0.806	0.293	−0.783	−0.949 to −0.316	0.007	−0.302	−0.777 to 0.398	0.396	−0.047	−0.654 to 0.596	0.897
	Type III Risk Stratification	8										−0.210	−0.793 to 0.562	0.618	−0.479	−0.888 to 0.310	0.230
Soft Tissue Infection (Skin/Ulcer/ Bone/Abscess/ Burns)	Type I + II Risk Stratification	11	0.623	0.052 to 0.888	0.041	0.344	−0.320 to 0.782	0.300	−0.760	−0.931 to −0.309	0.007	−0.296	−0.761 to 0.366	0.377	−0.055	−0.633 to 0.561	0.873
	Type III Risk Stratification	9										0.229	−0.504 to 0.770	0.554	−0.087	−0.702 to 0.605	0.824

*r*, Pearson correlation coefficient; CI, confidence interval; p, two-tailed nominal *p*-value; Appropriateness data missing for some months; DDD data complete *n* = 12 for all classes. 95% CI calculated via Fisher Z transformation: Z = 0.5 × ln[(1 + *r*)/(1−*r*)]; SE = 1/√(*n*−3); 95% CI for Z = Z ± 1.96 × SE; back-transformed to r. Computed using SPSS v29 CORRELATIONS/PRINT = TWOTAIL NOSIG CI (95)/MISSING = PAIRWISE. Correlation analysis was based on monthly aggregate observations, not individual prescriptions. *n* = number of monthly observations correlated with DDD. No adjustment for multiple comparisons was applied. All analyses are exploratory.

a Assisted by artificial Intelligence with data input and analysis were verified by the authors supported by statistician.

## 4 Discussion

The issue on the widespread use of third-generation Cephalosporins in Indonesia has been highlighted in a previous study (29). Other studies in Thailand and Cameroon have also highlighted the issue of third-generation Cephalosporins consumption being the most commonly prescribed antibiotics (30, 31). This might be due to physician's limited knowledge regarding antibiotics, inadequate laboratory facilities and poor institutional policies (31).

The WHO has documented that the third-generation Cephalosporins fall under the Watch group. Meanwhile, as has been explained earlier, WHO has set a target for prescribing antibiotic from the Access category as much as ≥60% of total antibiotic prescriptions. It is expected that the utilization of digital ASP tool may improve institutional policies for monitoring appropriate antibiotic consumption in hospitals. The digital ASP e-RASPRO tool has been implemented at the 10 hospitals since 2022.

Table 1 shows increased prescribing of initial empirical intravenous antibiotics that has been documented on the Digital ASP e-RASPRO tool. This may be due to several factors, such as an increase in the number of patients or improved compliance with the utilization of Digital ASP e-RASPRO tool that may lead to more physicians entering data into this digital device as a result of socialization efforts regarding its use that have been conducted in previous years. Table 1 also shows that the mean percentage on the appropriateness of initial intravenous empirical antibiotic consumption that had been increased from the first semester to the second semester. It is followed by Table 2, which illustrates a sharp increase in mean DDD of intravenous Penicillin-class antibiotics categorized as Access and a decrease in mean DDD of intravenous third-generation Cephalosporins-class antibiotics categorized as Watch group. It appears that there is a shift in the trend here. This may also be due to the socialization or awareness campaigns regarding the utilization of the tool that have been conducted in previous years that are associated with the WHO AWaRe initiatives.

The shift in prescribing trends toward antibiotics to the Access group (intravenous Penicillin) appears to be gaining momentum; however, there has also been increased consumption in other antibiotics classified as the Watch group, namely the intravenous Quinolone and Carbapenem classes. Regarding the increased antibiotic consumption, intravenous Quinolone and Carbapenem should be considered along with other biases such as increased number of patients in the 2nd semester, an increase in the number of patients classified in type III risk stratification group, or an increase in the number of patients with infections who are excluded from those with the four major infections, as well as an increase in the number of physicians prescribing intravenous Quinolones as an alternative antibiotic across all three stratification groups.

According to the antibiotic guideline in the Digital ASP e-RASPRO tool developed for the 10 hospitals, antibiotics from the Access group of the Penicillin class – such as Ampicillin Sulbactam and Amoxicillin Clavulanate in combination with Amikacin or Gentamicin—are indeed designated as antibiotics for patients in type I and II risk stratification groups. A previous study conducted at three hospitals in Indonesia found that more than 80% of patients fell into type I risk stratification group (32). If the same pattern holds true in this study, it could still influence a shift in antibiotic prescribing trends, particularly from the Watch Category to the Access Category.

According to the antibiotic guideline in the Digital ASP e-RASPRO tool, patients classified in the type I risk stratification group are empirically recommended to receive antibiotics from the Penicillin class (Ampicillin-Sulbactam or Amoxicillin-Clavulanate) with or without a combination of Aminoglycoside, namely Amikacin or Gentamicin. The intravenous third-generation Cephalosporins have been intentionally excluded from the list of antibiotics for patients in the type I risk stratification group in order to prevent a surge in prescriptions for intravenous third-generation Cephalosporins, which are classified as Watch Category antibiotics.

For the type II risk stratification group, empirical treatment guidelines recommend administering antibiotics from the Access Category of the Penicillin Class—specifically Ampicillin Sulbactam or Amoxicillin Clavulanate— which must be used in combination with an Aminoglycoside, such as Amikacin or Gentamicin. This combination is used as a Carbapenem Sparing Agent, as agreed upon by peer experts from the ten hospitals, to treat patient groups at higher risk of ESBL infections.

The agreed-upon antibiotic guidelines in the Digital ASP e-RASPRO tool has suggested that Carbapenem antibiotics should not be used empirically for patients in the type II risk stratification group (patients at risk for ESBL infections), with the aim of preventing a surge in empirical carbapenem prescriptions in daily practice.

Meanwhile, the Quinolone class antibiotic is still considered appropriate to be used as an alternative empirical treatment in all risk stratification groups, particularly in case of beta-lactam allergy or based on other clinical considerations. Since the same empirical antibiotics are used in both risk stratification groups, in Table 3 we have combined type I and II risk stratification group and correlated them with the mean DDD of each antibiotic class.

Table 3 The persistence of significant detrended correlations between the mean percentage on appropriate initial intravenous empirical antibiotics in the combined type I and II risk stratification group and the mean DDD of intravenous Penicillin (*r* = 0.598, 95% CI 0.021–0.873; *p* = 0.041) and intravenous third-generation Cephalosporins (*r* = −0.612, 95% CI −0.878 to −0.044; *p* = 0.035) provides robust evidence that e-RASPRO implementation is correlated with trend shifting from Watch to Access antibiotics beyond general time trends. The consistency of significant associations before and after detrending, with confidence intervals excluding trivial effects, indicates that e-RASPRO is associated with meaningful shifts toward WHO AWaRe-aligned prescribing behavior. Patient-level interrupted time-series analyses are warranted to confirm individual-level mechanisms. This study did not calculate the number of patients in each of the risk stratification groups (type I, II, and III); however, a previous study conducted at three hospitals in Indonesia using the Digital ASP e-RASPRO tool suggests that the number of patients in type I risk stratification group was significantly higher than those in type II and III risk stratification groups (32). If the same pattern occurs in this study, then the appropriateness of early intravenous empirical antibiotic consumption in type I risk stratification group will likely to have a greater influence on the correlation of the mean DDD of intravenous Penicillin with third-generation Cephalosporins antibiotics compared to those in type II risk stratification group.

The lack of a significant positive correlation between the mean percentage on the appropriateness of initial intravenous empirical antibiotic consumption in the combined type I and II risk stratification group for the four major infections and the mean DDD of Aminoglycoside antibiotics (*r* = 0.184, 95% CI −0.439–0.689; *p* = 0.568) may exist due to the fact that Aminoglycoside antibiotics has not been the preferred antibiotic of choice for physicians in daily clinical practice compared to penicillin antibiotics. In addition, Quinolone and Carbapenem are classified as Watch group antibiotics, in which a significant negative correlation is also expected. The Quinolone class can still be considered empirically appropriate for patients in type I and II risk stratification groups; however, its role has been marginalized as alternative antibiotic treatment.

The absence of a significant negative correlation between the mean percentage on the appropriateness of early intravenous empirical antibiotic consumption in the combined type I and II risk stratification group for the four major infections and the mean DDD of intravenous Quinolone antibiotics may be due to the fact that some physicians still consider prescribing intravenous Quinolone antibiotics as an alternative for patients classified in the combined type I and II risk stratification group based on specific clinical considerations, which can actually still be considered appropriate for them. However, the likelihood of using antibiotics from the intravenous Quinolone class here is not as high as that of those the intravenous Penicillin class. These findings may also indicate that the Penicillin class is the preferred class of antibiotics compared to the Quinolone class.

The consumption of intravenous Quinolone antibiotics for patients in type III risk stratification group, as well as the frequent switching of empirical antibiotic treatment or the switch into definitive antibiotic treatment using antibiotics from Quinolone class, may also be considered as potential causes that warrant further investigation. It may affect the mean DDD of quinolone antibiotics and influence the correlation among variables. Another possible reason for these results is the high number of cases of infections other than the ‘major four’ that are treated with Quinolone. Indonesia is a tropical country with many cases of typhoid fever or salmonellosis, for which the quinolone class is known as the primary empirical antibiotic of choice. These factors should be considered as potential influences on the mean DDD of Quinolone antibiotics.

The absence of a significant negative correlation between the mean percentage on the appropriateness of initial intravenous empirical antibiotic consumption in the combined type I and II risk stratification group for the four major infections and the mean DDD of Carbapenem-class antibiotics may also be influenced by the empirical carbapenem-class antibiotics consumption for patients in type III risk stratification group or other factors. Other reasons also need to be considered, such as the large number of clinicians prescribing carbapenem antibiotics based on culture results. However, more comprehensive research is needed to understand this more clearly.

Table 4 describes the correlation between the mean percentage on the appropriateness initial intravenous empirical antibiotic consumption in type III risk stratification group for the four major infections and the mean DDD of intravenous Quinolone and Carbapenem antibiotics. As previously explained, Carbapenem-class antibiotics, with or without combination therapy with Aminoglycoside-class antibiotics, are the recommended intravenous empirical antibiotic treatment in antibiotic guidelines mentioned the Digital ASP e-RASPRO tool for patients in the type III risk stratification group. Meanwhile, Quinolone-class antibiotics are used as alternative empirical antibiotic treatment in cases of Carbapenem allergy or based on other clinical considerations. Actually, these findings do not indicate a significant positive correlation between the appropriateness of initial intravenous empirical antibiotic consumption in type III risk stratification group for the four major infections and the mean DDD of intravenous Quinolone (*r* = 0.081, 95% CI −0.576–0.672; *p* = 0.825) and Carbapenem (*r* = −0.177, 95% CI −0.722–0.507; *p* = 0.625).

The utilization of Quinolone-class antibiotics in this group is still considered appropriate, even though Quinolone are considered as alternative antibiotics in this risk stratification group. Various analyses of other possibilities also need to be conducted, such as the potential use of quinolones in type I and II risk stratification groups as alternative antibiotic treatment or other factors that could affect the mean DDD of intravenous Quinolone.

A previous study using the Digital ASP e-RASPRO tool at three hospitals in Indonesia over a three-month period has shown that only about 14% of patients fall into type III risk stratification group III (32). If this were also the case in this study, then the number of patients requiring carbapenem-class antibiotics as initial empirical intravenous treatment would actually be quite small. This could certainly be a factor that may affect mean DDD of Carbapenem-class antibiotics. However, this certainly requires further research. However, it turns out that no significant correlation was found between the mean percentage on the appropriateness initial intravenous empirical antibiotic consumption in type III risk stratification group for the four major infections and the mean DDD of intravenous Quinolone and Carbapenem antibiotics, although the recommended antibiotic in this group of risk stratification is Carbapenem.

Table 5 provides robust statistical evidence that e-RASPRO implementation was correlated with shifting from Watch to Access antibiotics in infections where the shifting is guideline-concordant. The significant inverse correlation between the mean percentage on appropriate initial intravenous empirical antibiotics in the type I and II risk stratification group and the mean DDD of intravenous Carbapenem for intrabiliary infection (*n* = 9; *r* = −0.699, 95% CI −0.932 to −0.071; *p* = 0.036) reflects baseline empiric carbapenem use for type I and II risk stratification for intrabiliary infection was successfully de-escalated post e-RASPRO tool guidance.

In contrast, the mean percentage on the appropriateness of initial intravenous empirical antibiotics in the combined type I and II risk stratification group for intrabiliary infection was not correlated with the mean DDD of intravenous Penicillin and intravenous third-generation Cephalosporins; In this regard, various possibilities should be evaluated further.

In the pneumonia, urinary tract infection and pyelonephritis and soft tissue infection groups, there was a similar pattern to that described in Table 3. The results showed a significant positive correlation between suitability in type I and II risk stratification with DDD intravenous Penicillin and a significant negative correlation between suitability in type I and II risk stratification with DDD intravenous third-generation Cephalosporins.

In this manuscript, the DDD results may reflect broader hospital antibiotic use, including definitive therapy, subsequent antibiotic changes, and infections outside the four selected infection groups. This means the findings should remain exploratory and non-causal. Further investigation is also necessary regarding how many cases that need continuation of initial intravenous empirical antibiotics treatment to achieve patient recovery, or the treatment should be continued based on culture results, which would indicate that the initial intravenous empirical antibiotics administered are indeed effective in treating the patient. Certainly, such factors may also explain the correlation between the appropriateness of initial intravenous empirical antibiotic consumption and the mean DDD of antibiotics. This study does not control for several confounding factors, including patient volume, risk stratification distribution, case mix of infections, and infections other than the four major infections. Results must be interpreted cautiously. The analysis utilized 12-month aggregated data per infection type, and the potential for temporal confounding within each subgroup cannot be excluded, as demonstrated in the overall time-series sensitivity analysis. Therefore, these correlations should be considered hypothesis-generating rather than confirmatory of a causal relationship between e-RASPRO-guided appropriateness and antibiotic consumption patterns. Further research using more comprehensive methods is needed to establish causal relationships.

However, with these preliminary results, it is hoped that the utilization of the Digital ASP e-RASPRO tool can be an innovation in order antimicrobial stewardship program. Digital health technologies that can optimize the consumption of antimicrobial agents and can minimize the impact of healthcare on antimicrobial resistance have the potential to support development and utilization of high-quality data, which are highly beneficial for clinical management and improvement of decision-making (33).

## 5 Conclusion

Use of the e-RASPRO as a digital antimicrobial stewardship tool was correlated with increased intravenous Penicillin consumption and decreased third-generation Cephalosporins consumption. For patients stratified as risk types I and II, compliance was significantly correlated positively with intravenous Penicillin DDD and negatively with intravenous third-generation Cephalosporins DDD in pneumonia, urinary tract infections and pyelonephritis, and soft tissue infections. Further research using more comprehensive methods is needed to establish a causal relationship.

## Data Availability

The original contributions presented in the study are included in the article/Supplementary Material, further inquiries can be directed to the corresponding author.
